# An Efficient Cluster-Based Mutual Authentication and Key Update Protocol for Secure Internet of Vehicles in 5G Sensor Networks

**DOI:** 10.3390/s25010212

**Published:** 2025-01-02

**Authors:** Xinzhong Su, Youyun Xu

**Affiliations:** School of Communication and Information Engineering, Nanjing University of Posts and Telecommunications, Nanjing 210003, China; suxinz20@163.com

**Keywords:** Internet of Vehicles, security, mutual authentication, dynamic key update, low overhead

## Abstract

The Internet of Vehicles (IoV), a key component of smart transportation systems, leverages 5G communication for low-latency data transmission, facilitating real-time interactions between vehicles, roadside units (RSUs), and sensor networks. However, the open nature of 5G communication channels exposes IoV systems to significant security threats, such as eavesdropping, replay attacks, and message tampering. To address these challenges, this paper proposes the Efficient Cluster-based Mutual Authentication and Key Update Protocol (ECAUP) designed to secure IoV systems within 5G-enabled sensor networks. The ECAUP meets the unique mobility and security demands of IoV by enabling fine-grained access control and dynamic key updates for RSUs through a factorial tree structure, ensuring both forward and backward secrecy. Additionally, physical unclonable functions (PUFs) are utilized to provide end-to-end authentication and physical layer security, further enhancing the system’s resilience against sophisticated cyber-attacks. The security of the ECAUP is formally verified using BAN Logic and ProVerif, and a comparative analysis demonstrates its superiority in terms of overhead efficiency (more than 50%) and security features over existing protocols. This work contributes to the development of secure, resilient, and efficient intelligent transportation systems, ensuring robust communication and protection in sensor-based IoV environments.

## 1. Introduction

In recent years, the widespread deployment of sensor devices in various sectors, including urban transportation, healthcare, and the industrial Internet, has significantly improved connectivity and functionality [1,2]. In the Internet of Vehicles (IoV), a critical element of smart urban transportation, vehicles equipped with on-board units (OBUs) integrated with sensors and electronic control units (ECUs) exchange real-time data with external environments, enabling advanced functionalities such as autonomous driving and collision avoidance [3]. Furthermore, the external environment includes a wide array of roadside sensing devices, such as microwave radars, motion detection cameras, speed detection cameras, traffic light controllers, and dynamic display signs, which collectively form a comprehensive network [4,5,6,7,8,9,10]. At the core of this network are roadside units (RSUs), whose primary function is to collect and process real-time information on road conditions, traffic flow, and vehicle data. RSUs facilitate communication between vehicles, pedestrian devices, and other IoV nodes, including sensor networks and intelligent terminals, to enable end-to-end communication between vehicles, infrastructure, and pedestrians. This seamless data exchange between IoV entities is essential to improve road safety, traffic management, and overall system efficiency, while also raising significant security challenges due to the open and interconnected nature of these sensor-based networks [11,12,13,14].

Specifically, RSUs allow participating nodes to sense their surroundings by gaining access to operational data [15], traffic control information, congestion data, and visual blind spots of IoV devices within their range and authority. This expands their “field of view” to “see” vehicles, pedestrians, and buildings behind intersection masks, regardless of weather conditions. Information sharing among these participating entities can significantly reduce the incidence of traffic accidents and improve traffic safety [16,17].

With the global commercial launch of the 5th Generation Mobile Communication Technology (5G), 5G base station (gNodeB, gNB)-assisted communication is widely used in IoV and other systems due to its low latency, etc. [18]. In the 5G IoV system, information exchange between RSU and device, RSU and OBU, and OBU and OBU is connected through the 5G network [19,20], which can lead to various unknown risks. Due to the open nature of the channel, the attacker can intercept, replay, and falsify the transmitted information, and may launch multiple network attacks, resulting in system crashes. Therefore, it is crucial to ensure the confidentiality, integrity, and authenticity of IoV information transmission.

In recent years, authentication has been recognized as promising in protecting the security and privacy of nodes [21,22,23,24]. By verifying the identity of an entity, unauthorized access and message forgery can be eliminated and the confidentiality of transmitted data can be ensured. However, considering the diversity of attack types, most current relevant authentication schemes suffer from security flaws, and the overhead of the algorithms used is too high to be applicable for practical deployment in the IoV environment. Therefore, in this paper, we propose an efficient cluster-based mutual authentication and key update protocol (ECAUP) to realize low overhead and highly reliable information interaction among IoV nodes. For this paper, the main contributions are as follows:

The fine-grained access authority control of RSU to sensing devices is realized through the factorial tree, which must access the corresponding devices according to the group key generated in the registration phase, and low-cost encryption algorithms such as physical unclonable functions (PUFs) are utilized to provide physical layer security against device capture attacks while realizing end-to-end security authentication.

Considering the mobility of IoV and the device update of RSU clusters, a dynamic and flexible cluster key update scheme is proposed to provide perfect forward and backward secrecy for ECAP with anonymous and untraceable properties under the premise of security.

Verify the security of ECAUP’s session and update keys using tools such as BAN logic and Proverif, and analyze its reliability against various attacks and the security features it possesses. Moreover, the comparison with other schemes in terms of computation and communication overhead demonstrates the efficiency of the ECAUP.

Organization of the rest of the paper: Section 2 presents related research and work. Section 3 presents the mathematical background. Section 4 explains the authentication model and attacker model. Section 5 provides the implementation process of ECAUP. Section 6 provides a security analysis of ECAUP. Section 7 provides comparisons with other schemes. Section 8 summarizes the paper and gives future research directions.

## 2. Related Work

Currently, the related research on authentication in IOV has been continuously advancing. Xie et al. [25] proposed an authentication protocol based on blockchain and elliptic curve cryptography (ECC) to meet the needs of Vehicular Ad-hoc Networks (VANETs), which can realize the authentication between vehicle-to-vehicle and vehicle-to-roadside devices, and the method applies vehicle attributes, node pseudo-identity, and dynamic anonymity strategy within the protocol to ensure anonymity and untraceability. In addition, to achieve physical layer security, they use PUFs to cope with device capture attack and fuzzy-extractor-based bioinformation to avoid OBU intrusion attack.

Zhou et al. [26] proposed a mutual authentication protocol for vehicular sensor networks in 2017, but Wu et al. [27] found that the protocol could not solve the identity guessing attack, impersonation attack, and session key leakage. Therefore, Wu et al. proposed an efficient vehicle-to-vehicle secure communication authentication protocol to solve IoV security and privacy protection problems, which can realize secure mutual authentication between OBUs, and not only resist replay attack and guessing attacks but also ensure node anonymity. However, the scheme of Wu et al. cannot guarantee the forward secrecy of the communication session because it cannot address the impact of long-term key leakage.

Wang et al. [28] proposed a 5G-based end-to-end message authentication scheme for nodes applicable to VANETs that utilizes a group signature-based algorithm to achieve initial mutual authentication between vehicle-to-vehicle communication. In the proposed scheme, a vehicle first authenticates with a third-party trusted entity and uses one of the pseudo-identities to obtain its local signing private key, which can be used to sign messages to communicate with other vehicles in the neighborhood. In addition, to improve the computational speed of signature and modulo power operations, Wang et al. deployed a computational lookup table ahead of time in the registration phase, which improves the verification efficiency, and the related performance and security analyses demonstrate the effectiveness of the scheme in privacy protection.

Mun et al. [29] in 2022 proposed a new 5G-based Vehicle-to-Everything (V2X) security architecture that utilizes network slicing to enable V2X services with different characteristics and analyzes the security requirements of V2X services based on their ability to provide secure V2X authentication. The solution not only enables service authorization and revocation of participating nodes but also transmits information such as services and key credentials without revealing sensitive vehicle information. However, the computational overhead of the scheme proposed by Mun et al. is too high to meet the end-to-end latency requirements of autonomous driving in IoV.

Du et al. [30], in 2024, proposed an anonymous authentication protocol for 5G IoV nodes against impersonation attacks, which utilizes cryptographic algorithms such as elliptic curves to ensure the session privacy of the communication between OBU and RSU, and demonstrates its effectiveness against man-in-the-middle, replay, and other attacks through simulation and security analysis. The scheme uses temporary anonymized identity instead of the original identity and the nodes are unable to know each other’s real identity; hence, privacy is preserved. Moreover, the utilization of authentication to generate temporary key for communication eases the burden of key management and makes it difficult for attackers to obtain keys or tamper with messages.

However, the above schemes still have many flaws in terms of security and efficiency. For example, the overhead of the encryption method used is too high, and when actually deployed, the computation and communication overhead is too high to meet the low-latency requirement of IoV. Furthermore, all the above schemes lack access control on RSU authority, which makes them vulnerable to authority change attack from internal contaminated RSUs. Table 1 summarizes the related research of existing work.

## 3. Preliminaries

### 3.1. One-Way Hash Function

A one-way hash function [31] has an input and an output, where the input is called the message and the output is called the hash value. A one-way hash function can be calculated based on the content of the message hash value, and the hash value can be used to check the integrity of the message. Simply stated, the content of any length is converted into a fixed-length output string, and it is difficult to restore the original content through the output string. It is widely used in the fields of message summarization, message authentication code, key encryption, and data integrity verification. A one-way hash function can be denoted as $h:{\left\{0,1\right\}}^{*}\to {\left\{0,1\right\}}^{n}$, where $m\in {\left\{0,1\right\}}^{*}$ and $h\left(m\right)\in {\left\{0,1\right\}}^{n}$ are an input of arbitrary length binary strings and an output of fixed length binary strings, respectively.

### 3.2. Physical Unclonable Function

The Physical Unclonable Function [32,33] is a hardware security function based on physical characteristics. The PUF is a hardware security feature based on physical characteristics that takes advantage of uncontrollable differences in the manufacturing process of semiconductor devices to generate and store a unique identifier on a chip. The PUF generates a unique output for each access that is not stored on the chip and cannot be accessed, thus ensuring its unclonability.

PUF technology can be applied in many fields, such as information security, anti-counterfeiting authentication, Internet of Things (IoT), etc. (1) Information security: PUF can be used for key generation and storage. The unique identification generated by PUF can be used as the key generator in encryption systems to ensure the uniqueness and security of the key. (2) Anti-counterfeiting authentication: The PUF can be used for product anti-counterfeiting authentication. Each product can be equipped with a PUF, through the generation of unique identification, to achieve product anti-counterfeiting and traceability. (3) IoT: IoT devices need to be secure and trustworthy when deployed on a large scale. A PUF can be used for authentication and key generation of IoT devices to prevent them from being tampered with or cloned.

PUFs can be categorized into two types, i.e., strong PUFs and weak PUFs, based on factors such as the size of the chip. The number of challenge response pairs that can be generated by a strong PUF is much higher than that of a weak PUF, and usually, the researcher chooses the appropriate type of PUF based on the specific scenario. Suppose that we use a strong PUF in our scenario. The mathematical definition of a PUF can be denoted as PUF(C)=R, where *C* is the input incentive and challenge, and *R* is the output response. It is worth noting that since the PUF relies on the physical property of not storing any key, there may exist two responses, *R* and $R^{\prime }$, for the same input *C* in the actual authentication process. Usually, the threshold *r* is set and the authentication is passed when the Hamming distance between *R* and $R^{\prime }$ satisfies $HamDis(R,R^{\prime })\leq r$. The main purpose of the ECAUP utilizing a PUF is to reduce key storage and provide physical layer security. Therefore, in this paper, we do not consider the response difference of PUFs.

## 4. System Model

### 4.1. Authentication Model

**Figure 1:**
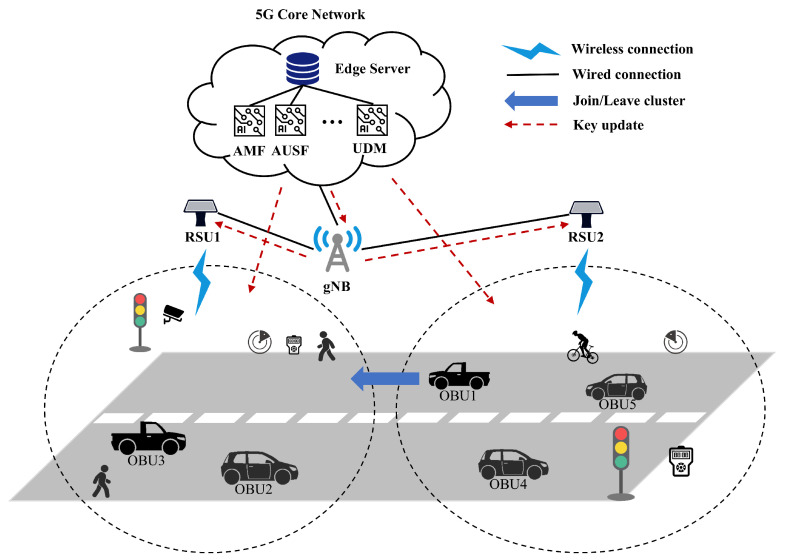
IOV authentication model.

As shown in Figure 1, the 5G IoV system mainly consists of a 5G core network (5GC) and a 5G radio access network (RAN). Distributing different types of IoV devices on both sides of the road, e.g., sensors such as speed camera, motion surveillance camera, and radar, as well as vehicles and pedestrians traveling on the road carrying a variety of intelligent terminals, e.g., OBU and cell phone, these intelligent terminals and devices constitute the so-called HIOV system. The 5GC-based system architecture includes various functions such as Access and Mobility Management Function (AMF), Authentication Server Function (AUSF), and Unified Data Management (UDM), which can be integrated into a particular ES. In the specific authentication process, the AMF is responsible for processing and providing 5G access, registration, and mobility management for participating nodes, including OBUs and roadside sensors. AUSF and UDM are responsible for maintaining the key management of the nodes, providing data upload and storage, and supporting authority modification configurations, authentication data, and subscription data. The 5G-enabled IoV mutual authentication protocol consists of four main components: RSU, ES, gNB, and IoV terminal device (IOVD).

Specifically, when there are collisions, traffic jams, fires, and other traffic conditions, various sensor devices in the IoV system will collect information related to traffic conditions and send it to RSU, which then forwards the information to vehicles and pedestrians in an end-to-end way, facilitating the traffic participants to make quick judgments, which indirectly expands the “traffic vision” of the drivers and pedestrians, and greatly decreases the number of traffic accidents. However, the communication radius of RSU is about 500–1000 m, to realize the access control of RSU to ensure the privacy of IOVD data. We divided the range of accessible devices for different RSUs. As shown in Figure 1, the two circles show the clusters of accessible devices for RSU1 and RSU2, respectively (note that the range is not only determined by the communication distance but also includes restrictions on the devices that can be accessed by the RSUs themselves).

When a new device, e.g., OBU1, wants to join the RSU1 cluster, the cluster key generated based on access control needs to be updated. Since the whole IoV system is a dynamic process, and the traditional offline key update method obviously does not meet the practical requirements, the key update needs to be realized in an online way.

In addition, we assume that ES is a completely trusted third-party entity, whose information such as internal keys cannot be captured by attackers. In contrast, RSUs and IOVDs are common participating nodes in the IoV system, which are not only susceptible to various network attacks but also have relatively simple internal hardware structures and very limited communication and computational resources compared to ES.

### 4.2. Threat Model

The security analysis model of our proposed scheme is based on an extension of the Dolev–Yao [34] threat model (DY model), in which some behaviors of an adversary can be defined:

(1) The adversary has the ability to generate random numbers and timestamps;
(2) The adversary may arbitrarily intercept, alter, delete, and relocate messages transmitted in the public channel;
(3) The adversary may attempt to impersonate and track nodes;
(4) The adversary may act as an intermediate node to forge and forward messages;
(5) The adversary may act as an insider attacker to change authorities;
(6) An adversary has the ability to physically capture devices;
(7) The adversary is unable to obtain any secret information from ES regardless of the method used.

## 5. Proposed Scheme

In this section, we detail the implementation process of ECAUP, which consists of five main phases as follows: (1) parameter setup, (2) IOVD registration, (3) RSU registration, (4) mutual authentication, and (5) dynamic key update. During the setup phase, some ECAUP public parameters (e.g., the type of PUF) are selected by a fully trusted ES. After setup, RSU and IOVD need to complete the registration with the help of ES. Then, a secure session key is generated between RSU and IOVD through mutual authentication for subsequent secure communication. Furthermore, ECAUP flexibly realizes online cluster key update when new/old devices join/exit. Table 2 lists the related symbols and their abbreviations used in ECAUP.

### 5.1. Parameter Setup Phase

Before authentication, there are three necessary operations performed by the ES. First, ES chooses a one-way hash function h(·) that generates a fixed-length string (e.g., hash-256), which guarantees the integrity and tamper-proofness of messages. Next, ES sets an appropriate PUF(·) for IOVD and stores a set of challenge and response pairs $\left(C_{i},R_{i}\right)$ generated from such PUF (assuming that a strong PUF is used) into its database, which can be used for subsequent authentication to provide physical-layer security. Finally, ES chooses a factorial tree architecture to generate cluster-based access authority key $C_{k}$ for RSU in the subsequent registration. Note: In ECUAP, we assume that ES has rich database functions such as store, find, modify and delete.

### 5.2. RSU Registration Phase

In general, IoV consists of many sensors, such as speed radar, localization device, motion detection camera, time detection camera, etc. However, the effective communication radius of RSU is about 500 m to 1000 m, which requires multiple RSUs to be controlled cooperatively to realize the interaction of the entire IoV sensor data. These RSUs can then interact with IOVDs to enable vehicles/pedestrians to “see” the complex road conditions behind the cover and react quickly, which greatly reduces the incidence of traffic accidents and improves the safety of IoV. Therefore, we achieve the authority attribution of RSU through fine-grained access control based on factorial tree [35] as follows:

(1) Factorial-tree-based fine-grained access control

The purpose of fine-grained access control is to authenticate and transmit information to IOVDs within range based on the communication radius of the RSU. However, due to the dynamic character of HIOV (i.e., the mobility of vehicles or pedestrians), the forward secrecy and backward secrecy of the key are difficult to guarantee. Thus, we provide online factorial-tree-based cluster key updates for ECAUP.

**Figure 2:**
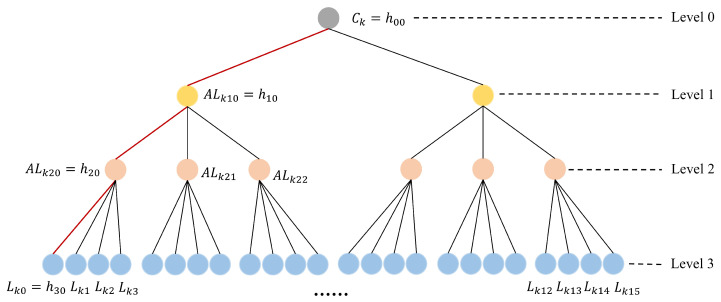
Factorial-tree-based accessible device table. The number of leaf nodes at each level in factorial tree is (t+1)!, where *t* is the level of the tree.

As shown in Figure 2, the cluster architecture of ECAUP can be simplified as a factorial tree, which consists of multiple layers of leaf nodes (level 1–level *t*)) and a single root node level 0, where *t* is the level of the tree. In the tree, layer *t* denotes the IOVDs accessible to RSU, i.e., $\left\{h_{t0}=L_{k0},h_{t1}=L_{k1},...,h_{t[(t+1)!-1]}=L_{k[(t+1)!-1]}\right\}$, where $\left\{L_{k0},L_{k1},...,L_{kt}\right\}$ is the leaf node key generated by ES to IOVD.

(1) $$h_{xy}=h\left\{h_{\left(x+1)(xy+2y\right)}∥,...,∥h_{\left(x+1)(xy+2y+x+1\right)}\right\}$$

Then, the auxiliary leaf node keys $AL_{kxy}=h_{xy}$ can be computed from (1), where x=1,2,...,t−1 and y=0,1,...,(x+1)!. Finally, the cluster key $C_{k}=h_{00}$ of RSU is computed.

(2) Registration

**Figure 3:**
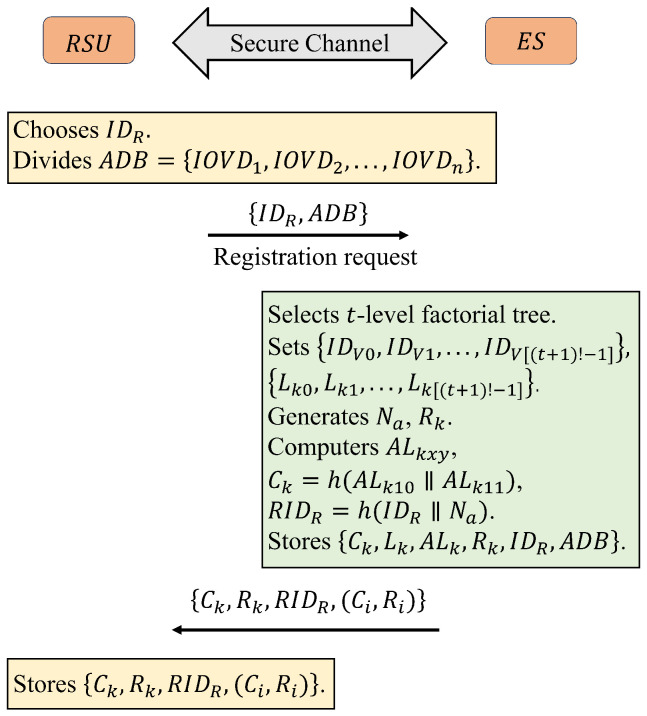
RSU registration.

First, RSU sends a registration request containing $ID_{R}$ and an accessible device table $ADT=\left\{IOVD_{1},IOVD_{2},...,IOVD_{n}\right\}$ to ES via a secure channel. Once the request from RSU is received, ES first selects an appropriate factorial tree level *t* based on the number of IOVDs *n*. ES then sets the identities $\left\{ID_{V0},ID_{V1},...,ID_{V[(t+1)!-1]}\right\}$ and leaf node keys $\left\{L_{k0},L_{k1},...,L_{k[(t+1)!-1]}\right\}$ for these devices and computes the corresponding auxiliary leaf node keys $AL_{kxy}$ and cluster/root key $C_{k}=h\left(AL_{k10}∥AL_{k11}\right)$. Next, ES also finds the corresponding device’s PUF-based challenge response pairs $\left(C_{i},R_{i}\right)$ in the database according to ADT. In addition, ES sets a master key $R_{k}$ for RSU and computes pseudo-identity $RID_{R}=h\left(ID_{R}∥N_{a}\right)$, where $N_{a}$ is a random number generated by ES. Finally, ES sends $\left\{C_{k},R_{k},RID_{R},\left(C_{i},R_{i}\right)\right\}$ to RSU via secure channel and stores $\left\{C_{k},L_{k},AL_{k},R_{k},ID_{R},ADT\right\}$ into its database. Upon receiving a message from the ES, the RSU stores the information contained in the message, such as the key, into memory as well. The RSU registration process is summarized in Figure 3.

### 5.3. IOVD Registration Phase

In the IOVD registration phase, a registration request containing $ID_{V}$ is first sent by IOVD to ES. The ownership of IOVD is then confirmed in the database by ES according to ADT. As shown in Figure 2, assuming that the level of the factorial tree is 3 and $h_{30}$ represents the current IOVD, ES selects the shortest path $\left\{h_{20},h_{10},h_{00}\right\}$ from the bottom up and fetches $\left\{L_{k0},AL_{k20},AL_{k10}\right\}$ in the database. Next, ES computes the pseudo-identity $RID_{V}=h\left(ID_{V}∥N_{b}\right)$, and $P=h(C_{k}∥ID_{R})$, where $N_{b}$ is a random number. Finally, ES sends $\left\{P,RID_{V},L_{k0},AL_{k20},AL_{k10}\right\}$ to IOVD in a secure channel and stores $ID_{V}$. Meanwhile, IOVD receives and stores the message from ES.

### 5.4. Mutual Authentication Phase

When the RSU wants to access the IOVD and obtain data, it needs to complete a two-way authentication operation between RSU and IOVD. After authentication is finished, a secure session key $Sk_{R}=Sk_{V}$ is established between RSU and IOVD for subsequent data transmission. Note that authentication is realized via an open channel (e.g., 5G core network/Internet) as follows:

Step 1: $RSU\to IOVD:\left\{N_{1}^{*},T_{1},RID_{R},C_{i}\right\}$

First RSU generates the random number $N_{1}$ and extracts the pair $\left(C_{i},R_{i}\right)$ from its memory according to the IOVD it wants to access, and then computes $N_{1}^{*}=N_{1}⊕h\left(RID_{R}∥h\left(C_{k}∥ID_{R}\right)\right)=N_{1}⊕TID_{R}$, $T_{1}=h(R_{i}∥RID_{R}∥h\left(C_{k}∥ID_{R}\right))$, where $TID_{R}$ is the temporary identity of RSU. Finally, $M_{1}=\left\{N_{1}^{*},T_{1},RID_{R},C_{i}\right\}$ is sent by the RSU to IOVD via public channel.

Step 2: $IOVD\to RSU:\left\{N_{2}^{*},T_{2},T_{3},T_{4},RID_{V}\right\}$

Once the message $M_{1}$ is received from RSU, IOVD extracts $N_{1}^{\prime }=N_{1}^{*}⊕h\left(RID_{R}∥P\right)$ to verify the freshness. If found fresh, the session continues; otherwise, it aborts. IOVD then inputs $C_{i}$ from $M_{1}$ into PUF to obtain the response $R_{i}$ and computes $T_{1}^{\prime }=h(R_{i}∥RID_{R}∥P)$ to verify $T_{1}^{\prime }\overset{?}{=}T_{1}$. If not satisfied, the session is terminated; otherwise, it continues. Next, IOVD generates the random number $N_{2}$, computes the temporary identity $TID_{V}=h\left(RID_{V}∥P\right)$, $N_{2}^{*}=N_{2}⊕TID_{V}$, $a_{i}=h\left(L_{k}∥ID_{V}\right)$, $T_{2}=N_{2}⊕a_{i}$, $T_{3}=h(P∥R_{i}∥N_{1}∥N_{2}∥ID_{V})$, $T_{4}=h(T_{3}∥a_{i}∥RID_{V})$, and sends $M_{2}=\left\{N_{2}^{*},T_{2},T_{3},T_{4}\right\}$ to RSU via public channel.

Step 3: $RSU\to IOVD:\left\{N_{3}^{*},T_{5},T_{6}\right\}$

When the message $M_{2}$ is received from IOVD, RSU extracts $N_{2}^{\prime }=N_{2}^{*}⊕h\left(RID_{V}∥h\left(C_{k}∥ID_{R}\right)\right)$ and verifies the freshness. If it is met, RSU further computes $a_{i}^{\prime }=T_{2}⊕N_{2}^{\prime }$, $T_{4}^{\prime }=h(T_{3}∥a_{i}^{\prime }∥RID_{V})$ and verifies $T_{4}^{\prime }\overset{?}{=}T_{4}$. When all the above conditions are met, the session continues; otherwise, it is terminated. RSU then generates a random number $N_{3}$ and computes $N_{3}^{*}=N_{3}⊕TID_{R}$, $b_{i}=h\left(R_{k}∥ID_{R}\right)$, $T_{5}=N_{3}⊕b_{i}$, $T_{6}=h(b_{i}∥N_{3}∥R_{i}∥RID_{R})$. Moreover, the ultimate session key $SK_{R}=h(a_{i}∥b_{i}∥T_{3}∥N_{3})$ is computed by RSU for subsequent communication. Finally, $M_{3}=\left\{N_{3}^{*},T_{5},T_{6}\right\}$ is sent by RSU to IOVD via a public channel.

**Figure 4:**
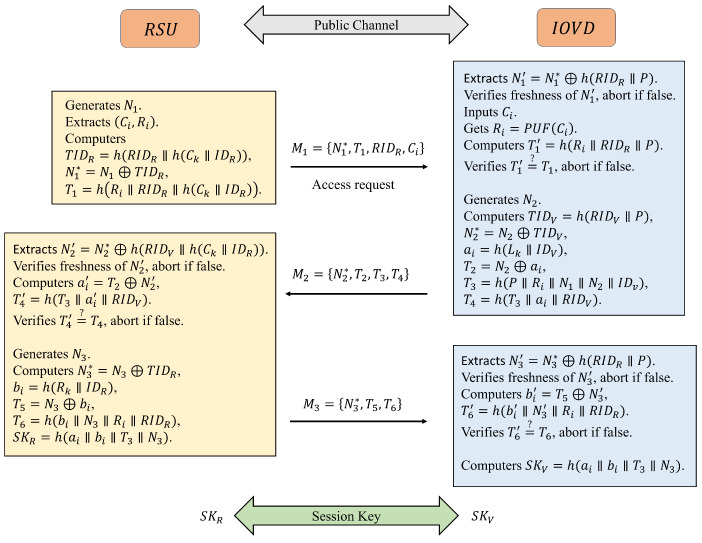
Mutual authentication between RSU and IOVD.

Step 4: Meanwhile, once $M_{3}$ is received, IOVD verifies the freshness of the random number through extraction $N_{3}^{\prime }=N_{3}^{*}⊕h\left(RID_{R}∥P\right)$. $N_{3}^{\prime }$ is determined fresh, and the session continues; otherwise, it terminates. IOVD then computes $b_{i}^{\prime }=T_{5}⊕N_{3}^{\prime }$, $T_{6}^{\prime }=h(b_{i}^{\prime }∥N_{3}^{\prime }∥R_{i}∥RID_{R})$ and verifies $T_{6}^{\prime }\overset{?}{=}T_{6}$. When both are equal, IOVD computes the session key $SK_{V}=h(a_{i}∥b_{i}∥T_{3}∥N_{3})=SK_{R}$, which is used for subsequent data encryption between RSU and IOVD. The authentication process is summarized in Figure 4.

### 5.5. Dynamic Key Update Phase

**Figure 5:**
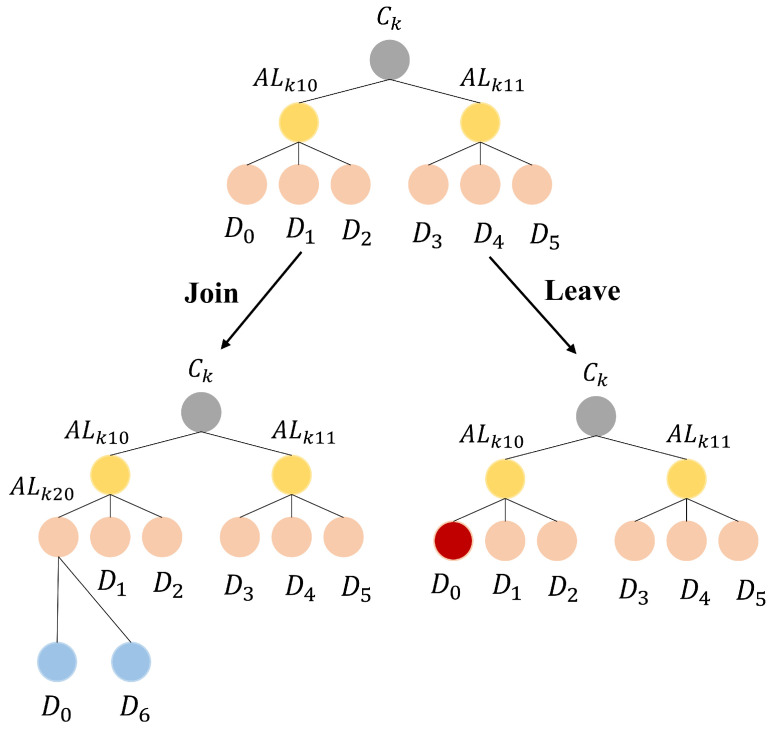
IOVD join and leave.

In this paper, an RSU can only access the corresponding sensor devices based on ADT. The accessible device table ADT needs to be kept up to date due to the mobility of participant nodes in urban transportation, e.g., sensors attached to pedestrians and vehicles. Moreover, for static sensors such as speed cameras/radar, ADT also needs to be updated when new/old devices join/leave the cluster of RSU. For ease of description, we assume that *t* is 2, as shown in Figure 5. Therefore, this section describes the dynamic update process of cluster key $C_{k}$ in ECAUP as follows.

The entire key update process of ECAUP is implemented in an online manner with the help of a fully trusted ES, which only requires that ES sends update messages unilaterally to IOVD and RSU, and does not involve multiple message interaction and authentication, which greatly reduces network congestion. First, ES resets the structure of the factorial tree and layer *t* according to the number of added devices, and generates the corresponding auxiliary leaf node keys $AL_{k}$ a and cluster key $C_{k}$. Then, depending on the forwarding logic rule of factorial tree, it implements the online update of Ck with the minimum number of messages.

(1) IOVD joins the cluster

ES→IOVD: ES first generates the random number $N_{j}$ and then computes the factorial tree element $AL_{k20}=h\left(L_{k0}∥L_{k6}\right)$, $AL_{k10}^{new}=h(AL_{k20}∥L_{k1}∥L_{k2})$, $C_{k}^{new}=h\left(AL_{k10}^{new}∥AL_{k11}\right)$, $P^{new}=h\left(C_{k}^{new}∥ID_{R}\right)$, the random number verification message $N_{j}^{*}=N_{j}⊕h\left(C_{k}∥ID_{R}\right)=N_{j}⊕P$, $N_{j}^{\prime *}=N_{j}⊕h\left(C_{k}^{pre}∥ID_{R}^{pre}\right)=N_{j}⊕P^{pre}$. Next, ES computes $\alpha_{1}=h(P∥AL_{k11}∥N_{j})$, $X_{1}=P^{new}⊕\alpha_{1}$ for $D_{3}$, $D_{4}$, $D_{5}$; $\alpha_{2}=h(P∥AL_{k10}∥N_{j})$, $X_{2}=P^{new}⊕\alpha_{2}$, $X_{3}=AL_{k10}^{new}⊕\alpha_{2}$, for $D_{1}$, $D_{2}$; $\alpha_{3}=h(P∥AL_{k10}∥L_{k0}∥N_{j})$, $X_{4}=P^{new}⊕\alpha_{3}$, $X_{5}=AL_{k10}^{new}⊕\alpha_{3}$, $X_{6}=AL_{k20}⊕\alpha_{3}$ for $D_{0}$; and $\alpha_{4}=h(P^{pre}∥L_{k6}∥N_{j})$, $X_{7}=P^{new}⊕\alpha_{4}$, $X_{8}=AL_{k10}^{new}⊕\alpha_{4}$, $X_{9}=AL_{k20}⊕\alpha_{4}$ for $D_{6}$. Finally ES broadcasts $Y_{1}=\left\{N_{j}^{*},X_{1}\right\}$, $Y_{2}=\left\{N_{j}^{*},X_{2},X_{3}\right\}$, $Y_{3}=\left\{N_{j}^{*},X_{4},X_{5},X_{6}\right\}$ and $Y_{4}=\left\{N_{j}^{\prime *},X_{7},X_{8},X_{9}\right\}$ to the devices in cluster. Finally, ES stores all the factorial tree information $C_{k}^{new}$, $AL_{k}^{new}$ into the database.

Upon receiving the broadcast messages, $D_{0}$,$D_{1}$,…,$D_{6}$ extract $P^{new}$ and the updated auxiliary leaf node keys $AL_{k}^{new}$, respectively. For instance, $D_{0}$ can only extract the secret information in $Y_{3}$ due to the absence of other path keys in the factorial tree. $D_{0}$ first extracts $N_{j}^{\prime }=N_{j}^{*}⊕P$ and verifies the freshness, and terminates the key update if it does not match. Then, $D_{0}$ extracts $P^{new}$, $AL_{k10}^{new}$, and $AL_{k20}$ to replace the original keys (the update process for $D_{1}$,$D_{2}$,…,$D_{6}$ follows in the same way). Therefore, dynamic key update can be perfectly achieved in ECAUP for the new IOVD join.

ES→RSU: ES generates a random number $N_{j1}$ and then computes $N_{j1}^{*}=N_{j1}⊕h\left(C_{k}∥ID_{R}\right)$, $S_{i}=h\left(R_{k}∥N_{j1}\right)$, $Z_{1}=C_{k}^{new}⊕S_{i}$, $Z_{2}=C_{i}^{new}⊕S_{i}$, $Z_{3}=R_{i}^{new}⊕S_{i}$ and sends $\left\{N_{j1}^{*},Z_{1},Z_{2},Z_{3}\right\}$ via an open channel to the RSU in the current cluster, where $C_{i}^{new}$ and $R_{i}^{new}$ are multiple challenges and responses from PUF of the new device taken out by ES in its database. $N_{j1}$ is first extracted and verified to be fresh when RSU receives the message from ES. Next, RSU extracts and stores $C_{k}^{new}$, $\left(C_{i}^{new},R_{i}^{new}\right)$ and deletes the original $C_{k}$, $\left(C_{i},R_{i}\right)$ in memory.

(2) IOVD leaves the cluster

As shown in Figure 5, the root key of the factorial tree also needs to be updated when the old device $D_{0}$ leaves the current cluster, which is similar to the joining process. First, ES changes the structure of the tree and computes the path key of the changed node (i.e., leaf node, auxiliary leaf node, root node) by hash, and then broadcasts messages to corresponding nodes through the factorial-tree-based forwarding logic rule. Meanwhile, RSU receives additional update messages from ES. Finally RSU and IOVDs verify the above messages and realize the update of the original key. Note that all dynamic key updates are accomplished via the public channel, regardless of the joining or leaving of devices.

## 6. Security Analysis

In this section, we verify the security of the ECAUP using both formal and informal security analysis. In particular, we first analyze the security of the session key generated between RSU and IOVD in the protocol utilizing BAN logic. Further, the robustness of the ECAUP in the face of various attacks is then given using informal security analysis. In addition, we perform a comprehensive verification of the protocol with Proverif, a popular automated verification tool.

### 6.1. BAN-Logic-Based Formal Security Analysis

In the reasoning process of BAN logic [36], the beliefs of participants in a protocol change continuously with the increase of message exchange. The application of BAN logic firstly requires an “idealization step”, i.e., converting the messages of the protocol into formulas in BAN logic; secondly, making reasonable assumptions based on the situation of the protocol, then reasoning according to the rules and assumptions; and, finally, deducing whether the protocol can accomplish the desired goal. In this paper, we verify the security of the session keys $SK_{R}$ and $SK_{V}$ generated in the ECAUP through BAN logic.

(1) Notation and rule

Some notations and semantics in BAN logic are as follows.

N1: P∣≡X: Principal *P* believes statement *X*.

N2: P⊲X: Principal *P* sees the statement *X*.

N3: P|∽X: Principal *P* once said the statement *X*.

N4: P⇒X: Principal *P* has jurisdiction over the statement *X*.

N5: #(X)/fresh(X): Formula *X* is fresh.

N6: ${\langle X\rangle }_{Y}$: A message synthesized from formula *X* and secret *Y*.

N7: $P\overset{K}{⟷}Q$: Formula *K* is a shared key of *P* and *Q*.

N8: $P\overset{X}{⇌}Q$: Formula *X* is a secret only known to *P* and *Q*.

N9: (X,Y): Formula (X,Y) containing formula *X* and formula *Y*.

N10: SK: Session key used in the current session.

BAN consists of 19 inference rules, and we only list the relevant rules used in ECAUP.

R1: $\frac{P|\equiv Q\overset{Y}{⇌}P,P⊲{\langle X\rangle }_{Y}}{P|\equiv Q|∽X}$: Message-meaning rule.

R2: $\frac{P|\equiv \#(X),P|\equiv Q|∽X}{P|\equiv Q|\equiv X}$: Nonce-verification rule.

R3: $\frac{P|\equiv \#(X)}{P|\equiv \#(X,Y)}$: Freshness rule.

R4: $\frac{P|\equiv Q\Rightarrow X,P|\equiv Q|\equiv X}{P|\equiv X}$: Jurisdiction rule.

R5: $\frac{P|\equiv (X,Y)}{P|\equiv X},\frac{P⊲(X,Y)}{P⊲X},\frac{P|\equiv Q|∽(X,Y)}{P|\equiv Q|∽X},\frac{P|\equiv Q|\equiv (X,Y)}{P|\equiv Q|\equiv X}$: Seeing and belief rule.

(2) Security goal

Based on the above BAN logic rules, the security goals of ECAUP can be expressed as follows:

G1: $RSU|\equiv (RSU\overset{SK}{⟷}IOVD)$.

G2: $IOVD|\equiv (RSU\overset{SK}{⟷}IOVD)$.

(3) Idealized form

In mutual authentication of ECAUP, messages $M_{1}$, $M_{2}$ and $M_{3}$ transmitted between RSU and IOVD can be abstractly represented as follows:

M1: $RSU\to IOVD:\left\{N_{1}^{*},T_{1},RID_{R},C_{i}\right\}$.

M2: $IOVD\to RSU:\left\{N_{2}^{*},T_{2},T_{3},T_{4},RID_{V}\right\}$.

M3: $RSU\to IOVD:\left\{N_{3}^{*},T_{5},T_{6}\right\}$.

M1: $RSU\to IOVD:({\langle N_{1},RID_{R},ID_{R}\rangle }_{C_{k}},{\langle RID_{R},C_{k},ID_{R}\rangle }_{R_{i}},RID_{R},C_{i})$.

M2: $IOVD\to RSU:({\langle N_{2},RID_{V},P\rangle }_{C_{k}},{\langle ID_{V},L_{k}\rangle }_{N_{2}},{\langle P,N_{1},N_{2},ID_{V}\rangle }_{R_{i}},{\langle P,N_{1},N_{2},ID_{V},L_{k},RID_{V}\rangle }_{R_{i}},RID_{V})$.

M3: $RSU\to IOVD:({\langle N_{3},RID_{R},ID_{R}\rangle }_{C_{k}},{\langle RID_{R},ID_{R}\rangle }_{N_{3}},{\langle R_{k},ID_{R},N_{3},RID_{R}\rangle }_{R_{i}})$.

(4) Assumption

Some assumptions related to the authentication process of ECAUP are listed according to the BAN logic.

A1: $RSU|\equiv \#(N_{2})$.

A2(a): $IOVD|\equiv \#(N_{1})$, A2(b): $IOVD|\equiv \#(N_{3})$.

A3: $RSU|\equiv IOVD\Rightarrow (N_{2},RID_{V},ID_{V},L_{k},P)$.

A4: $IOVD|\equiv RSU\Rightarrow (N_{1},N_{3},RID_{R},ID_{R},R_{k})$.

A5: $RSU|\equiv (N_{1},N_{3},ID_{R},R_{k},C_{i},R_{i},C_{k})$.

A6: $IOVD|\equiv (N_{2},ID_{V},L_{k},R_{i},C_{k})$.

A7: $RSU|\equiv (RSU\overset{C_{k}}{⇌}IOVD)$.

A8: $IOVD|\equiv (RSU\overset{C_{k}}{⇌}IOVD)$.

A9: $RSU|\equiv (RSU\overset{R_{i}}{⇌}IOVD)$.

A10: $IOVD|\equiv (RSU\overset{R_{i}}{⇌}IOVD)$.

(5) Security proof

Based on the above assumptions and the rules of BAN logic, we simplify the idealized form of ECAUP and provide the main proof procedure. Specifically, the IOVD receives an access request M1 and an authentication message M3 from RSU, both of which contribute to the realization of G2. According to M1, we can obtain the following information:

S1: $IOVD⊲({\langle N_{1},RID_{R},ID_{R}\rangle }_{C_{k}},{\langle RID_{R},C_{k},ID_{R}\rangle }_{R_{i}},RID_{R},C_{i})$.

S2: According to S1 and R5, we obtain $IOVD⊲{\langle N_{1},RID_{R},ID_{R}\rangle }_{C_{k}}$.

S3: According to A8 and R1, we obtain $IOVD|\equiv RSU|∽(N_{1},RID_{R},ID_{R})$.

S4: According to A2(a) and R3, we obtain $IOVD|\equiv \#(N_{1},RID_{R},ID_{R})$.

S5: According to S3, S4 and R2, we obtain $IOVD|\equiv RSU|\equiv (N_{1},RID_{R},ID_{R})$.

S6: According to S5, A4 and R4, we obtain $IOVD|\equiv (N_{1},RID_{R},ID_{R})$.

S7: According to S6 and R5, we obtain $IOVD|\equiv N_{1},IOVD|\equiv ID_{R}$.

According to M3, and repeating the above steps, we obtain

S8: $IOVD|\equiv R_{k},IOVD|\equiv N_{3}$.

S9: $IOVD|\equiv N_{2},IOVD|\equiv ID_{V},IOVD|\equiv L_{k},IOVD\left|\equiv R_{i},IOVD\right|\equiv C_{k}$.

S10: According to the session key $SK_{V}=h(a_{i}∥b_{i}∥T_{3}∥N_{3})=h(h\left(L_{k}∥ID_{V}\right)∥h\left(R_{k}∥ID_{R}\right)∥h(h\left(C_{k}∥ID_{R}\right)∥R_{i}∥N_{1}∥N_{2}∥ID_{V})∥N_{3})$ in ECAUP S7, S8, and S9, we can obtain G2: $IOVD|\equiv (RSU\overset{SK}{⟷}IOVD)$.

According to M2, we can obtain the following information:

S11: $RSU⊲({\langle N_{2},RID_{V},P\rangle }_{C_{k}},{\langle ID_{V},L_{k}\rangle }_{N_{2}},{\langle P,N_{1},N_{2},ID_{V}\rangle }_{R_{i}},{\langle P,N_{1},N_{2},ID_{V},L_{k},ID_{V}\rangle }_{R_{i}},RID_{V})$.

S12: According to S11 and R5, we obtain $RSU⊲{\langle P,N_{1},N_{2},ID_{V},L_{k},RID_{V}\rangle }_{R_{i}}$.

S13: According to A9 and R1, we obtain $RSU|\equiv IOVD|∽(P,N_{1},N_{2},ID_{V},L_{k},RID_{V})$.

S14: According to A1 and R3, we obtain $RSU|\equiv \#(P,N_{1},N_{2},ID_{V},L_{k},RID_{V})$.

S15: According to S13, S14 and R2, we obtain $RSU|\equiv IOVD|\equiv (P,N_{1},N_{2},ID_{V},L_{k},RID_{V})$.

S16: According to S15 and R5, we obtain $RSU|\equiv IOVD|\equiv (P,N_{2},ID_{V},L_{k},RID_{V})$.

S17: According to S16, A3 and R4, we obtain $RSU|\equiv (P,N_{2},ID_{V},L_{k},RID_{V})$.

S18: According to S17 and R5, we obtain $RSU|\equiv N_{2},RSU\left|\equiv ID_{V},RSU\right|\equiv L_{k}$.

S19: According to A5 and R5, we obtain $RSU|\equiv N_{1},RSU|\equiv N_{3},RSU|\equiv ID_{R},RSU|\equiv R_{k},RSU\left|\equiv R_{i},RSU\right|\equiv C_{k}$.

S20: According to the session key $SK_{V}=h(a_{i}∥b_{i}∥T_{3}∥N_{3})=h(h\left(L_{k}∥ID_{V}\right)∥h\left(R_{k}∥ID_{R}\right)∥h(h\left(C_{k}∥ID_{R}\right)∥R_{i}∥N_{1}∥N_{2}∥ID_{V})∥N_{3})$ in ECAUP S18 and S19, we can obtain G1: $RSU|\equiv (RSU\overset{SK}{⟷}IOVD)$.

The realizability and security of the session key $SK_{R}=SK_{V}$ generated between RSU and IOVD can be shown from S10 and S20.

### 6.2. Informal Security Analysis

RSU impersonation attack: In this attack, an adversary A attempts to impersonate $M_{1}=\left\{N_{1}^{*},T_{1},RID_{R},C_{i}\right\}$, $M_{3}=\left\{N_{3}^{*},T_{5},T_{6}\right\}$ and then serves as a legitimate node to communicate with IOVD, where $T_{1}=h(R_{i}∥RID_{R}∥h\left(C_{k}∥ID_{R}\right))$, $T_{5}=N_{3}⊕b_{i}$, $T_{6}=h(b_{i}∥N_{3}∥R_{i}∥RID_{R})$, $b_{i}=h\left(R_{k}∥ID_{R}\right)$. Even if A can generate the random numbers $N_{1}^{A}$ and $N_{3}^{A}$, there is no possibility to replace RSU as a real node due to the lack of critical secret information $R_{i}$, $C_{k}$, $ID_{R}$, and $R_{K}$. Thus, our scheme can cope with RSU impersonation attack.

IOVD impersonation attack: In ECAUP, IOVD sends only one authentication message $M_{2}=\left\{N_{2}^{*},T_{2},T_{3},T_{4}\right\}$, and A wants to fake $M_{2}$ and then communicate with RSU as a legitimate IOVD, where $T_{2}=N_{2}⊕a_{i}$, $T_{3}=h(P∥R_{i}∥N_{1}∥N_{2}∥ID_{V})$, $T_{4}=h(T_{3}∥a_{i}∥RID_{V})$, $a_{i}=h\left(L_{k}∥ID_{V}\right)$. However, A still cannot accomplish the impersonation attack (suppose A can generate random number) to IOVD due to the privacy of $L_{k}$, $ID_{V}$, and $R_{i}$. Therefore, our scheme is able to cope with the IOVD impersonation attack.

Replay attack: In this attack, A captures messages $M_{1}$, $M_{2}$, and $M_{3}$ and then replays them to RSU and IOVD, respectively, which in turn causes system crash. However, in ECAUP, replay attack initiated by A cannot work. For example, when IOVD receives the message $M_{1}=\left\{N_{1}^{*},T_{1},RID_{R},C_{i}\right\}$ replayed from A, it verifies the freshness of the random number and terminates the session immediately in case of non-compliance. Moreover, it is not possible for A to modify the random number $N_{1}$, due to the fact that $N_{1}^{*}=N_{1}⊕h\left(RID_{R}∥h\left(C_{k}∥ID_{R}\right)\right)=N_{1}⊕TID_{R}$ in $M_{1}$ is secretly encapsulated by the temporary identity $TID_{R}$. Similarly, $M_{2}$ and $M_{3}$ cannot pose a threat to RSU and IOVD even if they are replayed by A in the public channel. It is worth noting that the dynamic key update process in the ECAUP also uses random numbers. Therefore, our proposed protocol is not subject to replay attack.

IOVD Captured attack: In this case, A physically captures a certain IOVD to obtain all secret information $\left\{P,L_{k},AL_{k},ID_{V}\right\}$ inside it, and seeks to compute the session keys of the other devices from such information. Despite the fact that A possesses $\left\{P,L_{k},AL_{k},ID_{V}\right\}$, A cannot obtain the PUF-based challenge–response pairs $\left(C_{i},R_{i}\right)$. Assuming that A can generate the challenge $C_{i}^{\prime }$ and thus obtain response, the probability that adversary obtains $R_{i}$ is almost 0 due to the physically unclonable nature of PUF. Furthermore, in the ECAUP, different PUFs are assigned to IOVDs, and the secret information of each IOVD is completely different. Therefore, A cannot impose any threat to other devices through the captured IOVDs.

Man-in-the-middle attack: Suppose that A intercepts a message $M_{1}$ in an open channel, then generates a random number *n* and tries to compute $T_{1}^{\prime }=h(R_{i}∥RID_{R}^{\prime }∥h\left(C_{k}∥ID_{R}\right))$, $n^{\prime *}=n⊕h\left(RID_{R}^{\prime }∥h\left(C_{k}∥ID_{R}\right)\right)$. Subsequently, A sends a forged valid message $M_{1}^{\prime }=\left\{n^{\prime *},T_{1}^{\prime },RID_{R}^{\prime },C_{i}^{\prime }\right\}$ to IOVD, disabling it to confirm and distinguish whether or not the message has been modified. But, due to the absence of secret information $R_{i}$, $C_{k}$, and $ID_{R}$, it is virtually impossible for A to calculate legitimate message $M_{1}^{\prime }$ (similarly in the case of $M_{2}^{\prime }$ and $M_{3}^{\prime }$). Therefore, ECAUP can deal with the man-in-the-middle attack.

Stolen-verifier attack: In ECAUP, none of the participating nodes carries the storage verification table for assisted authentication. Therefore, our scheme is resistant to stolen-verifier attacks.

Authority modification attack: In this attack, an internal node RSU authorized by ES desires to access IOVDs beyond its own authority and obtains sensor data [37,38], which can be denoted as $ADT=\left\{IOVD_{1},...,IOVD_{n}\right\}$ to $ADT^{\prime }=\left\{IOVD_{1}^{\prime },...,IOVD_{n}^{\prime }\right\}$. According to Section 5.2, the access authority of RSU can be represented as $C_{k}$, which is based on a factorial tree hashed in a bottom-up manner. Suppose RSU computes a new cluster key $C_{k}^{R}$ using $ADT^{\prime }$ and the factorial tree, and sends an authentication request $M_{1}=\left\{N_{1}^{*},T_{1}^{R},RID_{R},C_{i}\right\}$ to IOVD, where $T_{1}^{R}=h(R_{i}∥RID_{R}∥h\left(C_{k}^{R}∥ID_{R}\right))$. Once the request is received, IOVD computes $T_{1}^{R^{\prime }}=h(R_{i}∥RID_{R}∥P)$ after verifying the freshness of $N_{1}$ and continues to verify $T_{1}^{R^{\prime }}\overset{?}{=}T_{1}^{R}$. If both differ, it indicates that the current RSU does not belong to its cluster and then terminates the session. The reason why the RSU cannot access devices outside of its authority is due to the fact that secret information $P=h(C_{k}∥ID_{R})$ is assigned by ES to all IOVDs in cluster as early as the registration phase. Thus, our proposed protocol can handle authority modification attacks.

Anonymity and untraceability: In the ECAUP, the messages $M_{1}=\left\{N_{1}^{*},T_{1},RID_{R},C_{i}\right\}$, $M_{2}=\left\{N_{2}^{*},T_{2},T_{3},T_{4}\right\}$ and $M_{3}=\left\{N_{3}^{*},T_{5},T_{6}\right\}$ generated from each authentication contain individual random numbers; thus, A cannot track participating nodes through $M_{1}$, $M_{2}$, and $M_{3}$. In addition, the ECAUP transmits messages using pseudo-identities $RID_{R}$ and $RID_{V}$ (public), and temporary identities $TID_{R}$ and $TID_{V}$ (private), which do not involve the original identities $ID_{R}$ and $ID_{V}$. Hence, our proposed protocol enables anonymity.

Known session key security: Since the session key $SK_{V}=h(a_{i}∥b_{i}∥T_{3}∥N_{3})=SK_{R}$ in each authentication contains secret information $\left\{L_{k},ID_{V},R_{k},ID_{R},P,R_{i}\right\}$ and random numbers $\left\{N_{1},N_{2},N_{3}\right\}$ of both current communicating parties, even if A obtains a certain session key, it cannot compute other session keys. Therefore, our protocol guarantees the security of the known session key.

Perfect forward secrecy and backward secrecy: In Section 5.5, a detailed key update process of the ECAUP is presented in a dynamic form. The purpose of the update is to ensure that the original RSUs can no longer access IOVDs that have left their clusters, thus guaranteeing the backward secrecy of the sensed data. Furthermore, assume that A obtains the long-term key and attempts to compute the session key to steal the previous information. However, it is difficult for A to obtain the cluster key $C_{k}$ in our system model due to the mobility of HIOV, resulting in keeping the key updated in real time. Therefore, it is obvious that ECAUP has perfect forward and backward secrecy.

### 6.3. Formal Verification with Proverif

Proverif is a formal verification tool [39] developed for automated reasoning about security properties in cryptographic protocols and automated analysis and verification of security protocols, which can support cryptographic primitives, including symmetric and asymmetric encryption, digital signature, hash function, bit commitment, and proof of signature for knowledge. In addition, Proverif can provide the formal path of attackers to breach the key, which enables researchers to improve the protocol. In IoV, communication between RSU and IOVD requires authentication. Therefore, we utilize Proverif to evaluate the security of ECAUP.

In this paper, three open channels, ch1, ch2, and ch3, and two secure channels, sch1 and sch2, are defined. The role of each channel is as follows: (1) RSU and ES complete the registration described in Section 5.2 and Section 5.3 via sch1; (2) IOVD and ES complete the registration described in Section 6.3 via sch2; (3) the public channel ch1 is used to realize mutual authentication between RSU and IOVD; (4) ES sends the key $P^{new}$ that needs to be updated as well as the auxiliary leaf node key $ALk^{new}$ to IOVD via ch2; (5) ES sends the new cluster/root key $Ck^{new}$ as well as the challenge–response pair $\left(C_{i}^{new},R_{i}^{new}\right)$ to RSU via ch3.

**Figure 6:**
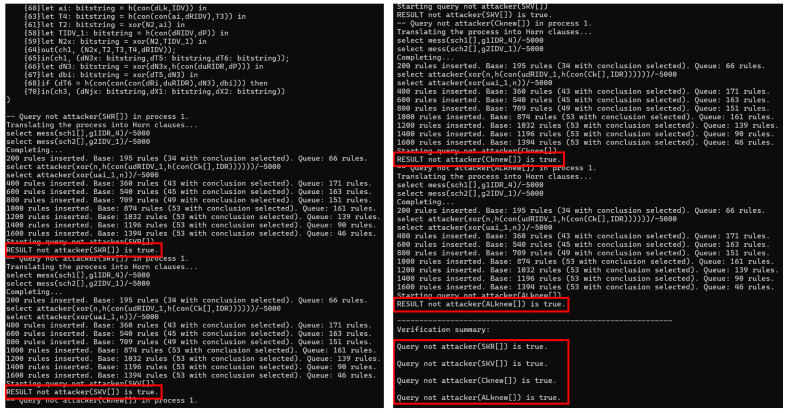
Proverif simulation results.

In the Proverif code, we define RSUauth, IOVDauth, and ES=ESReg1|ESReg2|ESupdate1|ESupdate2 to denote the sub-processes of RSU, IOVD, and ES, respectively, and, finally, the parallel execution of the three participating entities is realized through process(!RSUauth)|(!ES)|(!IOVDauth). From Figure 6, it can be inferred that the session keys $SK_{R}$ and $SK_{V}$, generated by RSU and IOVD authentication, and the update keys $Ck^{new}$ and $ALk^{new}$, sent by ES to RSU and IOVD, are secure. Thus, according to the simulation results, the robustness of ECAUP against various known network attacks is demonstrated.

## 7. Comparative Analysis

In this section, a comparison of the ECAUP with other schemes (Wu et al. [27], Wang et al. [28], Mun et al. [29] and Du et al. [30]) in terms of calculation cost, communication cost, and security features is given.

### 7.1. Calculation Costs Comparison

**Figure 7:**
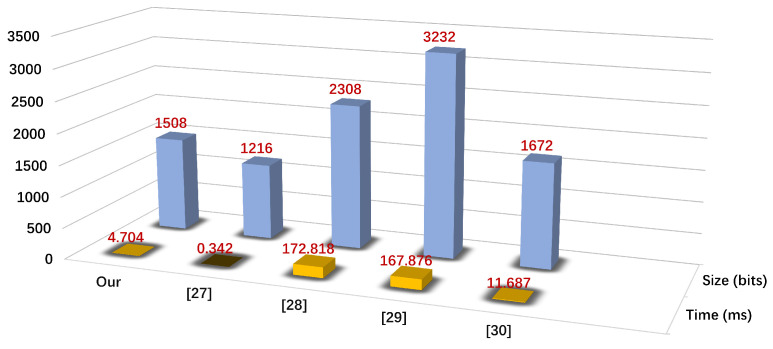
Comparison of communication cost and calculation cost.

We provide the computational cost required for the mutual verification phase of the ECAUP and other schemes. Assume that $T_{h}$, $T_{as}$, $T_{me}$, $T_{sig}$, $T_{bp}$, $T_{pm}$, $T_{pa}$, and $T_{puf}$ represent the time required for the hash function, symmetric encryption/decryption, modular exponentiation, digital signature/checksumption, bilinear pairing, ECC point multiplication, ECC point addition, and PUF response, respectively. According to the available experimental results [25,30,35], the time required to use these encryption operations are $T_{h}$ = 0.019 ms, $T_{as}$ = 19.536 ms, $T_{me}$ = 5.02 ms, $T_{sig}$ = 17.624ms, $T_{bp}$ = 44.517 ms, $T_{pm}$ = 2.61 ms, $T_{pa}$ = 0.576 ms, and $T_{puf}$ = 4.4 ms. From Table 3 and Figure 7, it can be observed that the cryptographic operation required for ECAUP authentication is $16T_{h}+T_{puf}$ and the computation time is 4.704 ms. Comparing to other schemes, it can be inferred that our protocol is suitable for end-to-end authentication in an IoV scenario because of the extremely low latency.

### 7.2. Communication Costs Comparison

To measure the communication costs of RSU and IOVD in the authentication phase, we assume that the output sizes of identity, public key, hash digest, random number, timestamp, PUF challenge and response, ECC point multiplication, digital signature, and symmetric encryption are 100 bits, 100 bits, 128 bits, 128 bits, 32 bits, 100 bits, 512 bits, 1024 bits, and 1024 bits, respectively. It can be deduced that the total communication cost of the ECAUP is 1508 bits, and the schemes of Wu et al. [27], Wang et al. [28], Mun et al. [29], and Du et al. [30] require 1216 bits, 2308 bits, 3232 bits, and 1672 bits, respectively. From Table 4 and Figure 7, it can be seen that our scheme requires only three handshakes to complete the authentication between RSU and IOVD, and requires only 1508 bits of communication cost. Therefore, the ECAUP is perfectly suitable for IoV scenarios.

### 7.3. Security Features Comparison

Table 5 shows the comparative analysis of the ECAUP with other schemes in terms of security and functional properties (abbreviations: ✓ support; ×: no support; —: not considered). It can be inferred that our scheme provides higher security and more properties compared to the other four protocols. It is worth noting that we not only consider the mobility of the participating nodes in IoV, but also the dynamic updating of the cluster key, which perfectly ensures the forward secrecy and backward secrecy of the whole cluster. Therefore, the ECAUP has wide prospects in the practical deployment of IoV.

## 8. Conclusions

In this paper, we proposed a lightweight end-to-end secure authentication and key update scheme for 5G-based IoV systems. The fine-grained access control RSU and subsequent dynamic key update forwarding method were provided via a factorial tree to ensure perfect forward and backward secrecy of the ECAUP. In addition, this paper used only low overhead algorithms such as hash to optimize the communication and computation overheads while guaranteeing the properties of node anonymity, non-traceability, and known session key security. The security analysis and comparison of other schemes showed that the ECAUP can meet the practical requirements of IoV. The focus of this paper is end-to-end authentication of IoV nodes, but the current key update overhead is high, so determining how to design and implement a more efficient and reliable key update protocol is one of the next research directions. Moreover, future research directions include the following: using the MIMT tool to implement STRIDE-based threat modeling for the ECAUP; session security analysis based on the ROR model; actual deployment of the ECAUP; and analyzing packet loss rate, latency, throughput, etc.

## Figures and Tables

**Table 1:** Related works.

Reference	Main Technologies Adopted	Advantages	Disadvantages
[25]	hash, ECC, PUF, blockchain, fuzzy extractor	OBU intrusion attack ✓ device capture attack ✓ MITM attack ✓ impersonation attack ✓ anonymity and untraceability ✓	privilege-insider attack × forward secrecy × known session key security × high resource cost × fine-grained access control ×
[26]	hash, asymmetric encryption	key update ✓ mutual authentication ✓	identity guessing attack × impersonation attack × device capture attack × session key disclosure × high resource cost × fine-grained access control ×
[27]	hash, asymmetric encryption	guessing attack ✓ session key security ✓ replay attack ✓ anonymity ✓	impersonation attack × forward secrecy × physical security × device capture attack × fine-grained access control ×
[28]	hash, digital signature, lookup table	privacy protection ✓ replay attack ✓ message integrity ✓ impersonation attack ✓ MITM attack ✓	privilege-insider attack × dynamic key update × physical security × anonymity and untraceability × high resource cost × fine-grained access control ×
[29]	hash, ECC, symmetric encryption	authorization and revocation ✓ privacy protection ✓ MITM attack ✓ privilege-insider attack ✓ anonymity and untraceability ✓	device capture attack × guessing attack × high resource cost × forward secrecy × fine-grained access control ×
[30]	hash, ECC	MITM attack ✓ replay attack ✓ forward secrecy ✓ impersonation attack ✓ anonymity and untraceability ✓	device capture attack × physical security × known session key security × high resource cost × fine-grained access control ×

**Table 2:** Notation and abbreviation.

Noation	Description
RSU, IOVD	Road side unit, IoV device
ES	Edge server
$ID_{R}$, $ID_{V}$	Identity of RSU and IOVD
$RID_{R}$, $RID_{V}$	Pseudo-identity of RSU and IOVD
$TID_{R}$, $TID_{V}$	Temporary identity of RSU and IOVD
ADT	Accessible device table of RSU
$C_{k}$	Cluster key of RSU
$L_{k}$	Leaf node key of IOVD
$AL_{1}$,…,$AL_{n}$	Auxiliary leaf node keys of IOVD
$R_{k}$	Master key of RSU
h(·)	One-way collision-resistant hash function
PUF(·)	Physical unclonable function
$C_{i}$	PUF-based challenge of IOVD
$R_{i}$	PUF-based response of IOVD
⊕, ‖	Bitwise XOR and concatenation operations
$N_{a}$, $N_{b}$	Random numbers for registration
$N_{1}$, $N_{2}$, $N_{3}$	Random numbers for authentication
$Sk_{R}$, $Sk_{V}$	Session keys shared between RSU and
	IOVD
A	Adversary
P→Q:M	*P* sends the message *M* to *Q*

**Table 3:** Calculation costs comparison.

Protocol	Authentication Total Cost	Time (ms)
[27]	$18T_{h}$	0.342 ms
[28]	$4T_{h}+5T_{me}+3T_{as}+2T_{bp}$	172.818 ms
[29]	$8T_{h}+2T_{me}+2T_{pm}+6T_{as}+2T_{sig}$	167.876 ms
[30]	$5T_{h}+4T_{pm}+2T_{pa}$	11.687 ms
Our	$16T_{h}+T_{puf}$	4.704 ms

**Table 4:** Communication costs comparison.

Protocol	Number of Messages	Size (Bits)
[27]	3	1216 bits
[28]	5	2308 bits
[29]	4	3232 bits
[30]	3	1672 bits
Our	3	1508 bits

**Table 5:** Security features comparison.

Feature	Our	[27]	[28]	[29]	[30]
RSU impersonation attack	✓	×	✓	—	✓
IOVD impersonation attack	✓	×	✓	✓	✓
Replay attack	✓	✓	✓	✓	✓
IOVD captured Attack	✓	×	×	×	×
Man-in-the-middle attack	✓	×	✓	✓	✓
Stolen-verifier attack	—	—	✓	—	—
Authority modification attack	✓	×	×	×	×
Insider privilege attack	✓	✓	×	✓	×
Off-line password guessing attack	✓	✓	✓	×	×
Anonymity and untraceability	✓	✓	×	✓	×
Known session key security	✓	✓	×	×	×
Perfect forward secrecy and backward secrecy	✓	×	✓	×	✓
Mutual authentication	✓	✓	✓	✓	✓
Key agreement	✓	×	✓	×	✓
Physical security	✓	×	×	×	×
Dynamic key update	✓	×	×	×	✓
Fine-grained access control	✓	—	—	—	—
Number of authentication messages	3	3	5	4	3

## Data Availability

Data are contained within the article.
